# Extraction optimization and influences of drying methods on antioxidant activities of polysaccharide from cup plant (*Silphium perfoliatum* L.)

**DOI:** 10.1371/journal.pone.0183001

**Published:** 2017-08-24

**Authors:** Hong-Mei Shang, Hai-Zhu Zhou, Ran Li, Meng-Ying Duan, Hong-Xin Wu, Yu-Jie Lou

**Affiliations:** 1 College of Animal Science and Technology, Jilin Agricultural University, Jilin, Changchun, China; 2 Key Laboratory of Animal Nutrition and Feed Science of Jilin Province, Jilin Agricultural University, Jilin, Changchun, China; 3 Grassland Research Institute of CAAS, Neimenggu, Hohhot, China; Institute of medical research and medicinal plant studies, CAMEROON

## Abstract

Response surface methodology (RSM) was used to investigate the extraction condition of polysaccharide from cup plant (*Silphium perfoliatum* L.) (named CPP). Water to raw material ratio (10–30 mL/g), extraction time (40–80 min) and extraction temperature (60–100°C) were set as the 3 independent variables, and their effects on the extraction yield of CPP were measured. In addition, the effects of drying methods including hot air drying (HD), vacuum drying (VD) and freeze drying (FD) on the antioxidant activities of CPP were evaluated. The results showed that the optimal condition to extract CPP was: water to raw material ratio (15 mL/g), extraction time (61 min), and extraction temperature (97°C), a maximum CPP yield of 6.49% was obtained under this condition. CPP drying with FD method showed stronger reducing power (0.943 at 6 mg/mL) and radical scavenging capacities against DPPH radical (75.71% at 1.2 mg/mL) and ABTS radical (98.06 at 1.6 mg/mL) than CPP drying with HD and VD methods. Therefore, freeze drying served as a good method for keeping the antioxidant activities of polysaccharide from cup plant. The polysaccharide from cup plant has potential to use as a natural antioxidant.

## Introduction

Cup plant (*Symphytum officinale* L.), belonging to genus *Silphium* L. of *Asteraceae* family, is a perennial herb native to North America. Cup plant has been used for medical purposes in North-American Indian tribes, such as the root of cup plant has nutritious and hidrotic properties [1]. The extracts of cup plant had antibacterial action towards *Enterococcus faecalis* and *Escherichia coli* [2].

Polysaccharide, widely distributing in animal, plant and microorganism, is a kind of polymeric carbohydrate [3]. Recently, natural polysaccharides have drawn much attention due to their biological activities, such as antitumor, antiviral action, immunomodulation, antilipidemic effect and oxidation resistance [4–6]. The extraction efficiency of polysaccharide from cup plant (CPP) has great signification for the development and utilization of this plant. However, there are few studies have been focused on the polysaccharide from cup plant. Extraction polysaccharide with hot water is the most common way used in food industry due to its easy accessibility and security [7]. Generally, extraction efficiency of polysaccharide is affected by multi factors, such as extracting time, extracting temperature and water to material ratio, and their influences on the polysaccharide yield may be independent or interactive. The response surface methodology (RSM) is widely applied to obtain the optimization extracting conditions of polysaccharide. The relationship among several variables can be investigated by RSM methodology.

In addition, the physicochemical properties and bioactivities of polysaccharide were significantly affected by its drying methods. Hot air drying, vacuum drying and freeze drying have been widely used for the drying process of polysaccharide. The most common drying method used in food processing is hot air drying due to its easy accessibility and lower cost. However, serious physicochemical changes of the dried polysaccharide may appear during hot air drying process [8]. The oxidization of the dried materials can be prevented during vacuum drying process. However, the structure damage of the dried products may occur under the high temperature of vacuum drying [9]. The bioactivities of the dried polysaccharides could be maximally protected under the vacuum and freeze condition during freeze drying process [10]. However, the cost of freeze drying is high because of the energy consumption during the long drying time needed. Therefore, the selection of drying methods is important for the maintaining of physicochemical characteristics and bioactivities of polysaccharides.

The aim of this study was to discover the potential of cup plant in production of CPP and to research the optimum extraction condition (water to raw material ratio, extraction time and extraction temperature) of CPP using RSM. In addition, the effects of three drying methods (hot air drying, vacuum drying and freeze drying) on the physicochemical properties and antioxidant activities of CPP were determined to seek the potential drying techniques. The *in vitro* antioxidant activities of CPP were evaluated on the base of 1,1-diphenyl-2-picrylhydrazyl (DPPH) radical scavenging activity, 2,2-azino-bis-3-ethyl-benzothiazoline-6-sulfonic acid) (ABTS) radical scavenging activity and ferric reducing power.

## Materials and methods

### Materials and reagents

Fresh cup plant was harvested in Jilin Agricultural University (Changchun, China). The plants were in bloom stage. The entire crop was cut into 5 cm segments before drying at 50°C, then the dried cup plant segments were ground into powders, and passed through a 1 mm sieve prior to extraction of the polysaccharide. The reagents including DPPH radical, ABTS radical and vitamin C were obtained from Sigma-Aldrich (St. Louis, USA). All other chemicals used were analytical grade and bought from local suppliers.

### Extraction of polysaccharide

The lipids of cup plant powder were taken out with 85% (v/v) ethanol for 24 h. The insoluble residue was dried in a vacuum freeze dryer and prepared for the extraction of polysaccharide with hot distilled water according to the designed water to raw material ratio, extraction time and extraction temperature. The extract was filtered with six layers gauze, and then the filtrate was concentrated to a 1/4 volume of the primary volume using a rotary evaporator under reduced pressure at 60°C. Subsequently, the concentrated solution was precipitated with 4 volumes of absolute ethanol. After being left for 12 h at 4°C, the precipitate was collected by centrifugation (3000 rpm for 15 min), and washed three times with absolute ethanol. Then the precipitate was dissolved with deionized water, removed protein by the Seveg reagent (4:1 of chloroform: normal butanol, v/v), and dialyzed in a dialysis bag (MWCO 1400 Da, Union Carbide). CPP was obtained by freeze-dried method. The extraction yield (*Y*) was calculated using the followed equation:

$$Y(\%)=\frac{W_{\mathrm{CPP}}}{W_{\mathrm{sample}}}\times 100$$ (1)

where *W*_CPP_ was the weight of CPP, and *W*_sample_ was the weight of cup plant powder used.

### Single-factor design

In the single-factor experiment, three variables including water to raw material ratio (10, 15, 20, 25 and 30 mL/g), extraction time (40, 50, 60, 70 and 80 min), and extraction temperature (60, 70, 80, 90 and 100°C) were taken into consideration to investigate their effects on the extraction yield of CPP. Each sample was extracted with hot distilled water according to the polysaccharide extraction procedure mentioned above. The CPP yields of different conditions were compared by one-way analysis of variance (ANOVA) using SPSS 17.0 (SPSS Inc., Chicago, IL).

### RSM design

After the appropriate ranges of water to raw material ratio (*X*_1_), extraction time (*X*_2_) and extraction temperature (*X*_3_) were achieved by the single-factor experiment. The Box–Behnken design (BBD, Design-Expert 8.0.6 Trial, State-Ease, Inc., Minneapolis, USA) was used to obtain the experiment design, statistic analysis result and regression model. Three variables of three levels each on the yield of CPP (*Y*) were performed. The whole design contained 17 experiment runs carried out in random order, and 5 central points were included in the experiment runs to measure the repeatability of the experiment method. The three levels (low, intermediate and high values) of each variable were encoded as −1, 0 and +1, respectively. For optimizing the extraction condition, a second-order polynomial formula was used to express the response (CPP yield) as a function of the variables:

$$Y=A_{0}+\sum_{i=1}^{3}A_{i}X_{i}+\sum_{i=1}^{3}A_{ii}X_{i}^{2}+\sum_{i=1}^{2}\sum_{j=i+1}^{3}A_{ij}X_{i}X_{j}$$ (2)

where *Y* is the dependent variable (CPP yield); *A*_0_, *A*_*i*_, *A*_*ii*_ and *A*_*ij*_ are the regression coefficients of constant coefficient, linear coefficient, quadratic coefficient, and interaction terms of two variables, respectively; *X*_*i*_ and *X*_*j*_ are the independent variables; *X*_*ij*_ is the interaction term, and *X*_*i*_^2^ is the quadratic term.

### Drying procedure of CPP

CPP was extracted under the optimum extraction condition, and dried using three different methods including hot air drying (HD), vacuum drying (VD) and freeze drying (FD). HD was carried out in an electric heating air-blowing drier (101-2-BS, Shanghai Yuejin Medical Instrument co., LTD, Shanghai, China) at 50°C. VD was done in a vacuum drying oven (DZF, Shanghai Longyue Instrument Equipment co., LTD, Shanghai, China) at 50°C. FD was carried out in a vacuum freeze dryer (SCIENTZ-12N, Ningbo Scientz Biotechnology co., LTD, Ningbo, China) at −70°C. The CPP obtained by HD, VD and FD were named as HD-CPP, VD-CPP and FD-CPP, respectively.

### Physicochemical properties of CPP

#### Chemical composition analysis

The total polysaccharide content of CPP was determined by phenol-sulfuric acid method using D-glucose as standard [11]. Uronic acid content of CPP was measured by *m*-hydroxybiphenyl assay using glucuronic acid as reference material [12]. Protein content of CPP was measured by Bradford’s method using bovine serum albumin as standard [13]. Sulfate radical content of CPP was determined by barium chloride-gelatin nephelometry using potassium sulfate as standard [14].

#### Moisture, pH and relative viscosity determinations

The moisture content of the CPP was measured according to the literature [15]. The relative viscosity of CPP (10 mg/mL) to deionized water was determined using a rotation viscometer (NDJ-8S, Shanghai Jitai Electronic Technology co., LTD, Shanghai, China) at 25°C. The pH of the CPP (2 mg/mL) was measured using a pH meter (PHSJ-5, Shanghai Yidian Scientific Instrument co., LTD, Shanghai, China).

#### Solubility test

The solubility of CPP was determined in distilled water. Briefly, the CPP sample (0.1 g) was placed into distilled water (50 mL) in a beaker, and then the beaker was placed in a water bath of different temperature (20, 40, 60, 80, 100°C). The CPP solution was stirred (150 rpm/min) with a digital mixer (JJ-5, Medical Instrument Factory of Jintan City, Jintan, China) until it was completely dissolved, and the length of dissolved time was recorded.

#### Complex formation with Congo red

The CPP sample (1.0 mg) was dissolved in deionized water (2.0 mL), and then aqueous Congo red (2.0 mL, 80 μmol/L) in 0.001 M NaOH was added to the CPP solution. Aqueous solution of NaOH (4.0 mol/L) was added to the mixture till the final concentration of NaOH in the mixture was in the range from 0 to 0.5 mol/L. The maximum absorption wavelength (λ _max_) of the reaction solution was measured using a spectrophotometer (752, Shanghai Xianke Spectrophotometer Instrument co., LTD, Shanghai, China).

### Assay of antioxidant activities of CPP *in vitro*

#### DPPH radical scavenging activity

DPPH radical scavenging activity of CPP was measured according to a previous method with some modifications [16]. Vitamin C was used as the positive control. Briefly, CPP were dissolved in distilled water to obtain a series of concentrations (0.1, 0.2, 0.4, 0.6, 0.8, 1.0 and 1.2 mg/mL). Then the CPP solution (3.0 mL) was mixed with 1.0 mL of 0.1mM DPPH solution in ethanol. The absorbance of mixture was determined at 517 nm after incubating at room temperature for 30 min in the dark environment. The ability of CPP to scavenge the DPPH radical was calculated according to the following equation:

$$\mathrm{DPPH}\;\mathrm{radical}\;\mathrm{scavenging}\;\mathrm{activity}\;(\%)=\frac{[A_{0}-(A_{1}-A_{2})]\times 100}{A_{0}}$$ (3)

where *A*_0_ is the absorbance of the control (3.0 mL distilled water and 1.0 mL of DPPH); *A*_1_ is the absorbance of the CPP sample; *A*_2_ is the absorbance of the CPP sample under the same condition to *A*_1_ except ethanol instead of the DPPH.

#### ABTS radical scavenging activity

ABTS radical scavenging activity of CPP was determined with a previous method with minor modifications [17]. CPP were dissolved in distilled water to obtain a series of concentrations (0.2, 0.4, 0.6, 0.8, 1.0, 1.2, 1.4, 1.6 and 1.8 mg/mL). In order to produce the ABTS radical cation, the reaction of 5 mL of ABTS solution (7 mM) and 1 mL of K_2_S_2_O_8_ aqueous solution (15 mM) was performed at 20°C overnight in the dark. To obtain the absorbance about 0.70 (±0.02) at 734 nm, the ABTS^+^ solution was diluted with deionized water. Then the CPP solution (0.75 mL) was mixed with 3 mL of the ABTS^+^ solution, and the mixture was reacted for 15 min at 20°C. After reaction, the absorbance at 734 nm was measured. Vitamin C was used as the positive control. The ABTS radical scavenging activity of CPP was calculated according to the equation below:

$$\mathrm{ABTS}\;\mathrm{radical}\;\mathrm{scavenging}\;\mathrm{activity}\;(\%)=\frac{[A_{0}-(A_{1}-A_{2})]\times 100}{A_{0}}$$ (4)

where *A*_0_ is the absorbance of the control group (water instead of CPP in the reaction system); *A*_1_ is the reaction result of polysaccharide sample; *A*_2_ is the absorbance of the sample only (ABTS solution was instead with water).

#### Ferric reducing power

The ferric reducing power of CPP was evaluated with the reported method [18] with minor modifications. The CPP samples were dissolved in distilled water to obtain different concentrations (1, 2, 3, 4, 5 and 6 mg/mL). Then, 1.5 mL of CPP solution was mixed with 1.5 mL of sodium phosphate buffer (0.2 M, pH 6.6) and 1.5 mL of K_3_[Fe(CN)_6_] (1%, w/v). The mixture was incubated at 50°C for 20 min. Subsequently, the mixture was cooled to room temperature, and then 1.5 mL of Cl_3_CCOOH (TCA, 10%, w/v) was added. After centrifugation at 3000 rpm for 10 min, 1.5 mL of the supernatant was taken out and mixed with 1.5 mL of deionized water and 0.3 mL of FeCl_3_ (0.1%, w/v). After reaction for10 min, the absorbance at 700 nm was measured. Vitamin C was served as the positive reference reagent. The ferric reducing power of CPP was calculated using the formula below:

$$\mathrm{Ferric}\;\mathrm{reducing}\;\mathrm{power}=A_{1}-A_{2}$$ (5)

where *A*_1_ is the absorbance of the CPP sample; *A*_2_ is the absorbance of the CPP sample only (distilled water instead of FeCl_3_ solution).

### Statistical analysis

All experiments were performed at least three times. Analyses of all samples were run in triplicate and averaged. All values were presented as mean±standard deviation (SD). The statistical analysis was performed using one-way ANOVA in SPSS 17.0 for Windows (SPSS Inc., Chicago, IL). Duncan post hoc tests were performed when significant differences were found. *P* < 0.05 was considered to be significant, and *P* < 0.01 was regarded as highly significant. The effects of CPP concentrations on the indexes of *in vitro* antioxidant activity assay were evaluated using curve estimation for linear and quadratic terms.

## Results and discussion

### Single-factor experiments for CPP extraction

#### Effect of water to raw material ratio on the yield of CPP

**Fig 1:**
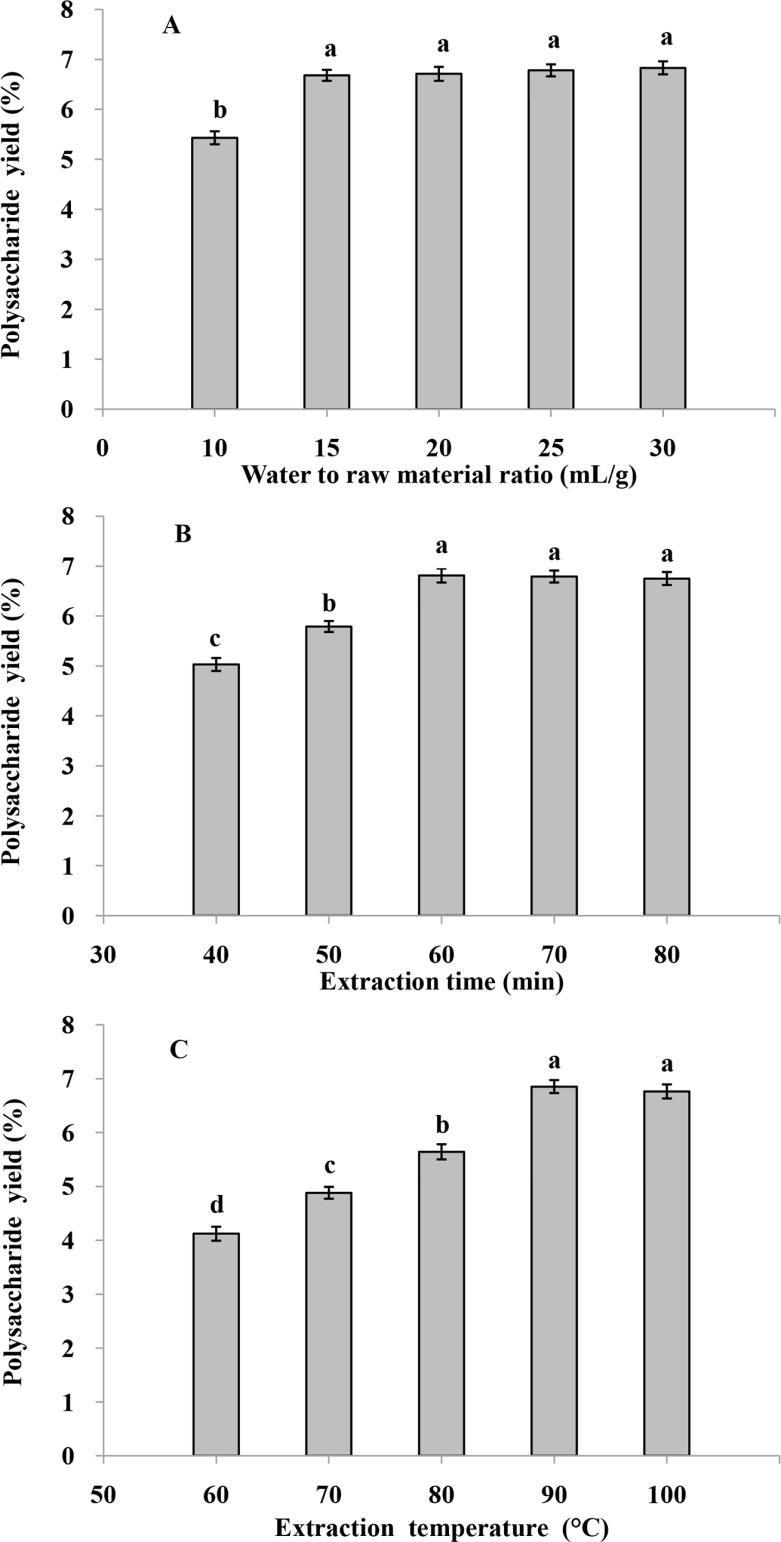
**Effects of (A) water to raw material ratio, (B) extraction time and (C) extraction temperature on the polysaccharide extraction yield of cup plant.** Different letters in the same bar chart indicate significant difference (*P* < 0.05).

To evaluate the influence of water to raw material ratio on the extraction yield of CPP (Fig 1A), the polysaccharide was extracted at different water to raw material ratio (10, 15, 20, 25 and 30 mL/g), while the extraction time and temperature were fixed at 60 min and 90°C, respectively. The extraction yield of CPP increased from 5.43% to 6.71% accompanying the increase of water to raw material ratio from 10 to 15 mL/g, and the CPP yield of 15 mL/g was significant higher than that of 10 mL/g (*P* < 0.05). However, when the water to raw material ratio continued to increase, there was no significance in the extraction yield of CPP (*P* > 0.05). The phenomenon might be explained by the difference of concentration between the interior tissue of cup plant and the exterior solvent [19]. As the water to raw material ratio increasing, the dissolution of polysaccharide from the interior tissue of material significantly improved, and the extraction yield of CPP increased. However, along with the further improvement of water to raw material ratio, the diffusion distance of polysaccharide from the intra-tissue increased and caused an insignificant increase of CPP yield [20]. Therefore, the water to raw material ratio of 15 mL/g was chosen as the central point of BBD experiment.

#### Effect of extraction time on the yield of CPP

Extraction time is an important parameter affecting the extraction yield of polysaccharide. To investigate the influence of extraction time on the extraction yield of CPP (Fig 1B), the polysaccharide was extracted at different extraction time (40, 50, 60, 70 and 80 min), and the water to raw material ratio and extraction temperature were set as 15 mL/g and 90°C, respectively. The extraction yield of CPP significantly increased from 5.03% to 6.81% along with the increase of extraction time from 40 to 60 min (*P* < 0.05). When the extraction time increased further, the extraction yield of CPP reduced slightly. The reason might be due to the degradation of polysaccharide under excessive extraction time at high extraction temperature condition [21]. Therefore, the extraction time of 60 min was selected as the central point of BBD experiment.

#### Effect of extraction temperature on the yield of CPP

To evaluate the influence of extraction temperature (60, 70, 80, 90 and 100°C) on the yield of CPP (Fig 1C), the water to raw material ration and extraction time were fixed at 15 mL/g and 60 min, respectively. The extraction yield of CPP increased from 4.12% to 6.85% when the temperature increased from 60 to 90°C, and the CPP yield of 90°C was significantly higher than those of 60, 70 and 80°C (*P* < 0.05). However, the extraction yield of CPP at 100°C was slightly lower than that of 90°C (*P* > 0.05). The reasons might be due to the diffusion coefficient of CPP from plant tissue increased with the improvement of temperature, but too high extraction temperature, such as extracting in boiling water solution, might cause the structural degradation of CPP [22]. Therefore, the extraction temperature of 90°C was selected as the central point of BBD experiment.

### Optimization of CPP extraction by Box-Behnken design

#### Prediction model

Base on the single-factor experiments, 17 runs were performed to optimize the extraction conditions of CPP. The designed matrix and the corresponding CPP yields (%) of the 17 runs to evaluate the 3 independent variables including water to raw material ratio (*X*_1_), extraction time (*X*_2_) and extraction temperature (*X*_3_) were demonstrated in Table 1.The CPP yields were in the range of 5.16% to 6.93%. Multiple regression analysis on the experiment values was performed, and the response model *Y* was described by the second-order polynomial Eq (6) as follow:

$$\begin{array}{l} Y(\%)=-79.16+0.940X_{1}+0.749X_{2}+1.22X_{3}-0.0005X_{1}X_{2}-0.002X_{1}X_{3}+0.0002X_{2}X_{3}\\ -0.022X_{1}{}^{2}-0.006X_{2}{}^{2}-0.007X_{3}{}^{2} \end{array}$$ (6)

where *Y* is the CPP yield; *X*_1_, *X*_2_ and *X*_3_ are the variables of water to raw material ratio, extraction time and extraction temperature, respectively.

**Table 1:** Box-Behnken experiment design and the response values for the extraction yield of CPP.

Run	Water to raw material ratio (*X*_1_) (mL/g)	Extraction time (*X*_2_) (min)	Extraction temperature (*X*_3_) (°C)	CPP yields (%)	
				Actual value	Predicted value
**1**	-1(10)	-1(50)	0(90)	5.26	5.32
**2**	1(20)	-1(50)	0(90)	5.63	5.68
**3**	-1(10)	1(70)	0(90)	5.70	5.65
**4**	1(20)	1(70)	0(90)	5.97	5.91
**5**	-1(10)	0(60)	-1(80)	5.16	5.17
**6**	1(20)	0(60)	-1(80)	5.70	5.72
**7**	-1(10)	0(60)	1(100)	5.74	5.73
**8**	1(20)	0(60)	1(100)	5.81	5.80
**9**	0(15)	-1(50)	-1(80)	5.32	5.25
**10**	0(15)	1(70)	-1(80)	5.45	5.50
**11**	0(15)	-1(50)	1(100)	5.58	5.53
**12**	0(15)	1(70)	1(100)	5.78	5.85
**13**	0(15)	0(60)	0(90)	6.75	6.81
**14**	0(15)	0(60)	0(90)	6.81	6.81
**15**	0(15)	0(60)	0(90)	6.71	6.81
**16**	0(15)	0(60)	0(90)	6.93	6.81
**17**	0(15)	0(60)	0(90)	6.85	6.81

CPP, polysaccharide from cup plant.

The result of ANOVA analysis was shown in Table 2. The significance of coefficients could be check by the *P*-values, and a smaller *P*-value indicated a more significant of the corresponding coefficient. As present in Table 2, the model *P*-value was less than 0.0001, which indicated that the regression model for CPP yield was significant. In addition, the *P*-values of the linear coefficients (*X*_1_, *X*_2_ and *X*_3_), and the quadratic term coefficients (*X*_1_^2^, *X*_2_^2^ and *X*_3_^2^) were lower than 0.01, which indicated the highly significant effects of these items on the extraction yield of CPP. The *P*-value of the interaction term coefficient (*X*_1_*X*_3_) was found to be lower than 0.05, indicating the significant influence of this item on the extraction yield of CPP. The other term coefficients (*X*_1_*X*_2_, *X*_2_*X*_3_) were not significant (*P* > 0.05). The ANOVA results indicated that water to raw material ratio (*X*_1_), extraction time (*X*_2_) and extraction temperature (*X*_3_) were significant single variables affecting the extraction yield of CPP.

**Table 2:** ANOVA analysis for the response surface quadratic model of CPP extraction.

Source	Sum of squares	df	Mean square	*F*-value	*P*-value
**Model**	5.88	9	0.65	79.97	< 0.0001**
***X***_**1**_	0.20	1	0.20	23.87	0.0018**
***X***_**2**_	0.16	1	0.16	19.65	0.0030**
***X***_**3**_	0.20	1	0.20	24.84	0.0016**
***X***_**1**_***X***_**2**_	2.554E-003	1	2.554E-003	0.31	0.5937
***X***_**1**_***X***_**3**_	0.058	1	0.058	7.11	0.0322*
***X***_**2**_***X***_**3**_	1.341E-003	1	1.341E-003	0.16	0.6976
***X***_**1**_^**2**^	1.28	1	1.28	156.30	< 0.0001**
***X***_**2**_^**2**^	1.62	1	1.62	197.66	< 0.0001**
***X***_**3**_^**2**^	1.82	1	1.82	222.48	< 0.0001**
**Residual**	0.057	7	8.176E-003		
**Lack of fit**	0.027	3	9.093E-003	1.21	0.4126
**Pure error**	0.030	4	7.489E-003		
**Cor Total**	5.942	16			
***R***^**2**^	0.9904				
**Adj *R***^**2**^	0.9780				
**C.V%**	1.52				
**Adeq Precision**	23.709				

*P values < 0.05 were considered to be significant.

**P values <0.01 were considered to be highly significant.

CPP, polysaccharide from cup plant.

The determination coefficient (*R*^2^) of the model was 0.9904, indicating that only 0.96% of total variations could not explain by the model. Additionally, the adjusted determination (adj-*R*^2^) value (0.9780) indicated most of the CPP yield variation could be predicted by the regression model. The coefficient of variation (C.V.) was at low value (1.52%), indicating the experiment data and predicated data were similar. According to the experimental data, the lack of fit *P*-value (0.4126) was more than 0.05, suggesting that the lack of fit was insignificant relating to the pure error. Adeq Precision was applied to evaluate the signal to noise ratio [23]. The Adeq Precision (23.709) in the present model was an adequate signal, which indicated that the model could be used to navigate the design space.

#### Response surface plot

**Fig 2:**
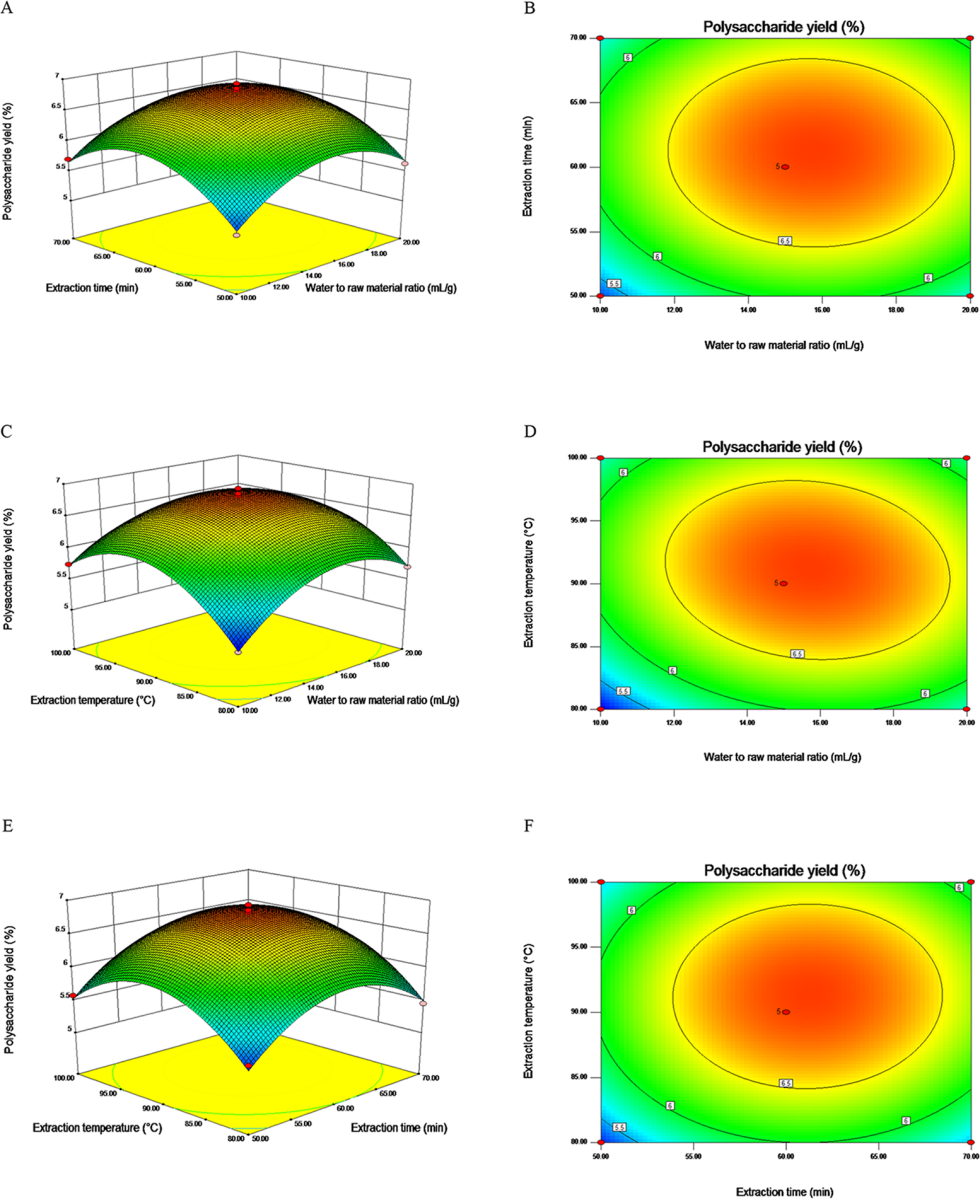
Response surface and contour plots for the polysaccharide yield of cup plant: effects of (A and B) water to raw material ratio and extraction time; (C and D) water to raw material ratio and extraction temperature on the polysaccharide yield; (E and F) extraction time and extraction temperature on the polysaccharide yield.

The visual interpretation of individual and interaction effects of variables on the CPP yield could be presented with 3-dimensional (3D) response surface plots and their corresponding 2-dimensional (2D) contour plots. A variable was set at the 0 level, and anther two variables were described in the 3D and 2D surface plots. The patterns of the 2D contour plots could indicate the mutual interactions significance between two tested variables. The contour plot of circular shape indicates insignificant mutual interactions between the two variables, while the contour plot of elliptical or saddle shapes suggests significant mutual interactions between the two variables [24]. For water to raw material ratio and extraction time (Fig 2B), the contour plot is circular, which indicated that the mutual interaction between water to raw material ratio and extraction time was not significant (*P* > 0.05). The similar trend (Fig 2F) was found for extraction time and extraction temperature (*P* > 0.05). The elliptical contour plot (Fig 2D) indicated the mutual interaction between water to raw material ratio and extraction temperature was significant (*P* < 0.05). These results were in agreement with the values in Table 2.

#### Optimization of the regression model

By analyzing the regression equation, the optimal conditions for the extraction of CPP were obtained as follow: water to raw material ratio 15.29 mL/g, extraction time 61.25 min, and extraction temperature 97.39°C. A maximum predicted CPP yield of 6.58% was obtained under these conditions. Considering the convenience during actual operation, the actual extraction conditions were modified slightly: water to raw material ratio 15 mL/g, extraction time 61 min, and extraction temperature 97°C. To verify the adequacy of the optimal condition, 3 verification tests were carried out and the extraction yield of CPP was found to be 6.49±0.08% (n = 3), which was in good agreement with the predicted CPP yield of 6.58%. Therefore, the regression model was adequate for predicting the extraction conditions of CPP.

### Physicochemical properties of CPP obtained by different drying methods

#### Yield and chemical composition

The yields and bioactivities of polysaccharides are significantly influenced by the type of drying and drying temperature used. The yields and chemical composition of the three CPP were shown in Table 3. The yield of FD-CPP (6.46%) was significantly higher than those of HD-CPP (5.87%) and VD-CPP (5.92%) (*P* < 0.05), indicating that freeze drying serves as a better method for the drying of polysaccharide from cup plant. The bioactivities of polysaccharides are affected by their chemical composition and molecular conformation [25]. Therefore, it was necessary to analyze the contents of chemical composition in CPP samples. As it was shown in Table 3, the contents of total polysaccharide, protein, uronic acid and sulfate radical in HD-CPP, VD-CPP, and FD-CPP were increased from 57.54% to 59.41%, 0.58% to 1.14%, 2.47% to 3.57%, and 1.28% to 1.81%, respectively. The contents of total polysaccharide, protein and uronic acid in FD-CPP were significantly higher than those of HD-CPP and VD-CPP (*P* < 0.05). These might due to the degeneration of the chemical constituents at the temperature of 50°C during hot air drying and vacuum drying. It was reported that there was a significant correlation between the radical scavenging activity and the uronic acid content of tea polysaccharide [26]. Therefore, the higher content of uronic acid might suggest the higher antioxidant capacities of FD-CPP.

**Table 3:** The yields and chemical composition of CPP obtained by different drying methods.

Samples	HD-CPP	VD-CPP	FD-CPP
**Polysaccharide yield (%)**	5.87 ± 0.06^b^	5.92 ± 0.05^b^	6.46 ± 0.04^a^
**Total polysaccharide content (%)**	57.54 ± 0.26^c^	58.64 ± 0.23^b^	59.41 ± 0.12^a^
**Protein content (%)**	0.58 ± 0.04^c^	0.72 ± 0.04^b^	1.14 ± 0.07^a^
**Uronic acid content (%)**	2.47 ± 0.07^c^	2.87 ± 0.06^b^	3.57 ± 0.08^a^
**Sulfate radical content (%)**	1.28 ± 0.12^b^	1.78 ± 0.09^a^	1.81 ± 0.10^a^
**Moisture content**	10.73 ± 0.44^a^	10.28 ± 0.32^a^	10.25 ± 0.27^a^
**pH**	7.22 ± 0.02^a^	7.23 ± 0.02^a^	7.23 ± 0.03^a^
**Relative viscosity**	1.03 ± 0.01^b^	1.14 ± 0.05^b^	1.42 ± 0.10^a^

Values are expressed as mean ± standard deviation (n = 3).

Means within a row with different superscripts (a-c) differ significantly (*P*<0.05).

CPP, polysaccharide from cup plant; HD-CPP, polysaccharide obtained by hot air drying; VD-CPP, polysaccharide obtained by vacuum drying; FD-CPP, polysaccharide obtained by freeze drying.

#### Moisture, pH and relative viscosity of CPP

The moisture contents, pH and relative viscosity of the three CPP were shown in Table 3. There were no significant differences in moisture contents of HD-CPP (10.73%), VD-CPP (10.28%) and FD-CPP (10.25%), as well as pH of HD-CPP (7.22), VD-CPP (7.23) and FD-CPP (7.23) (*P* > 0.05). The property of low moisture contents of CPP is a good fit for large scale application in food industry. The relative viscosity of FD-CPP (1.42) was significantly higher than those of HD-CPP (1.03) and VD-CPP (1.14) (*P* < 0.05), indicating that freeze drying method was a better method for drying the polysaccharide from cup plant.

#### Solubility of CPP

**Fig 3:**
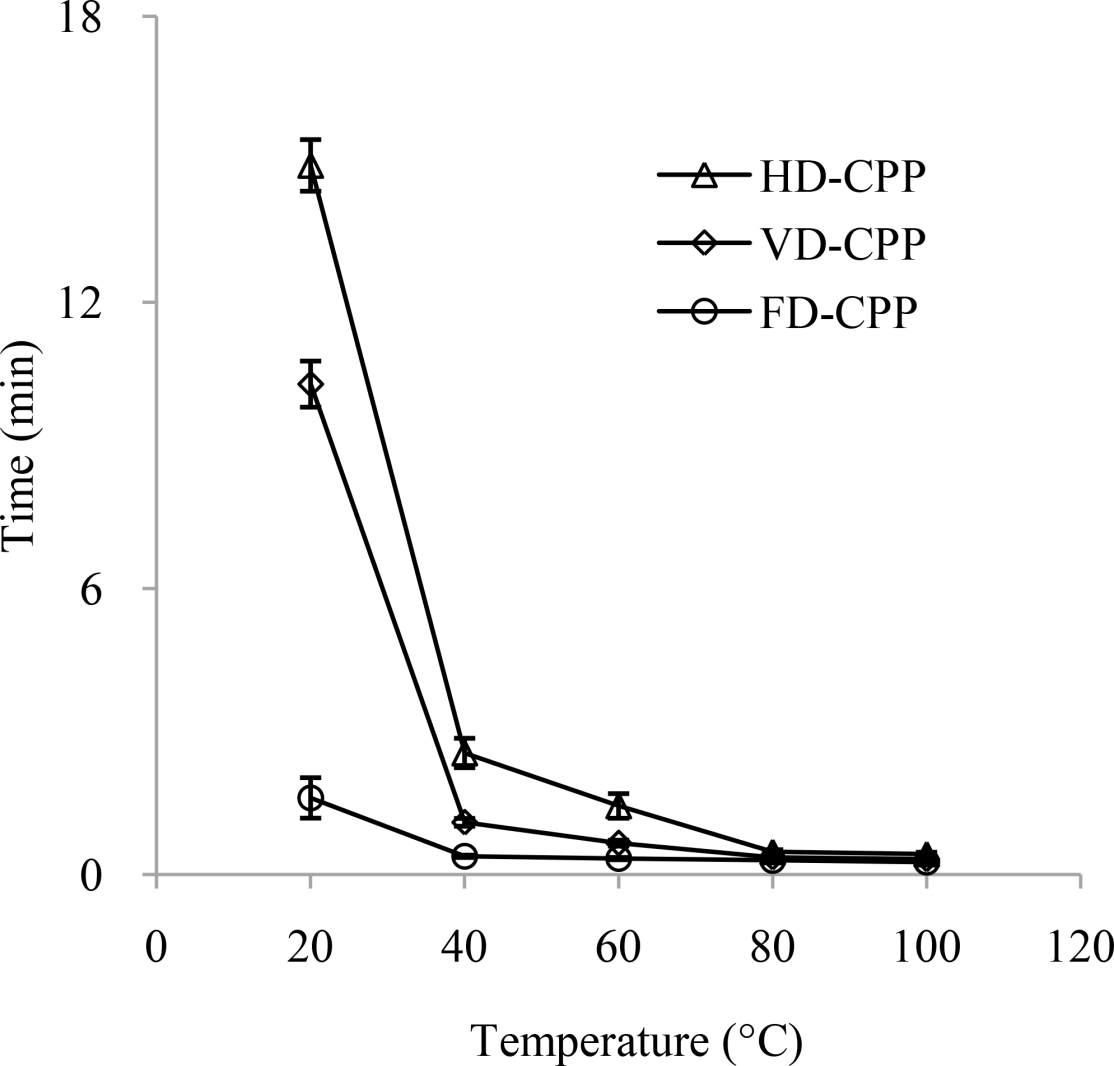
Solubility of cup plant polysaccharide obtained from three drying methods. CPP, polysaccharide from cup plant; HD-CPP, polysaccharide obtained by hot air drying; VD-CPP, polysaccharide obtained by vacuum drying; FD-CPP, polysaccharide obtained by freeze drying.

The solubility of CPP was shown in Fig 3. The dissolved time for CPP was gradually reduced as the temperature increasing. At the temperature of 20 and 40°C, the time for FD-CPP to dissolve was significantly lower than those of polysaccharides obtained by the other two drying methods (*P* < 0.05). The dissolved time of FD-CPP was also shorter than those of HD-CPP and VD-CPP at 60, 80 and 100°C. The reason might be due to that the looser structure of FD-CPP made it easier to combine with water during the process of re-dissolving.

#### Complex formation with Congo red of CPP

**Fig 4:**
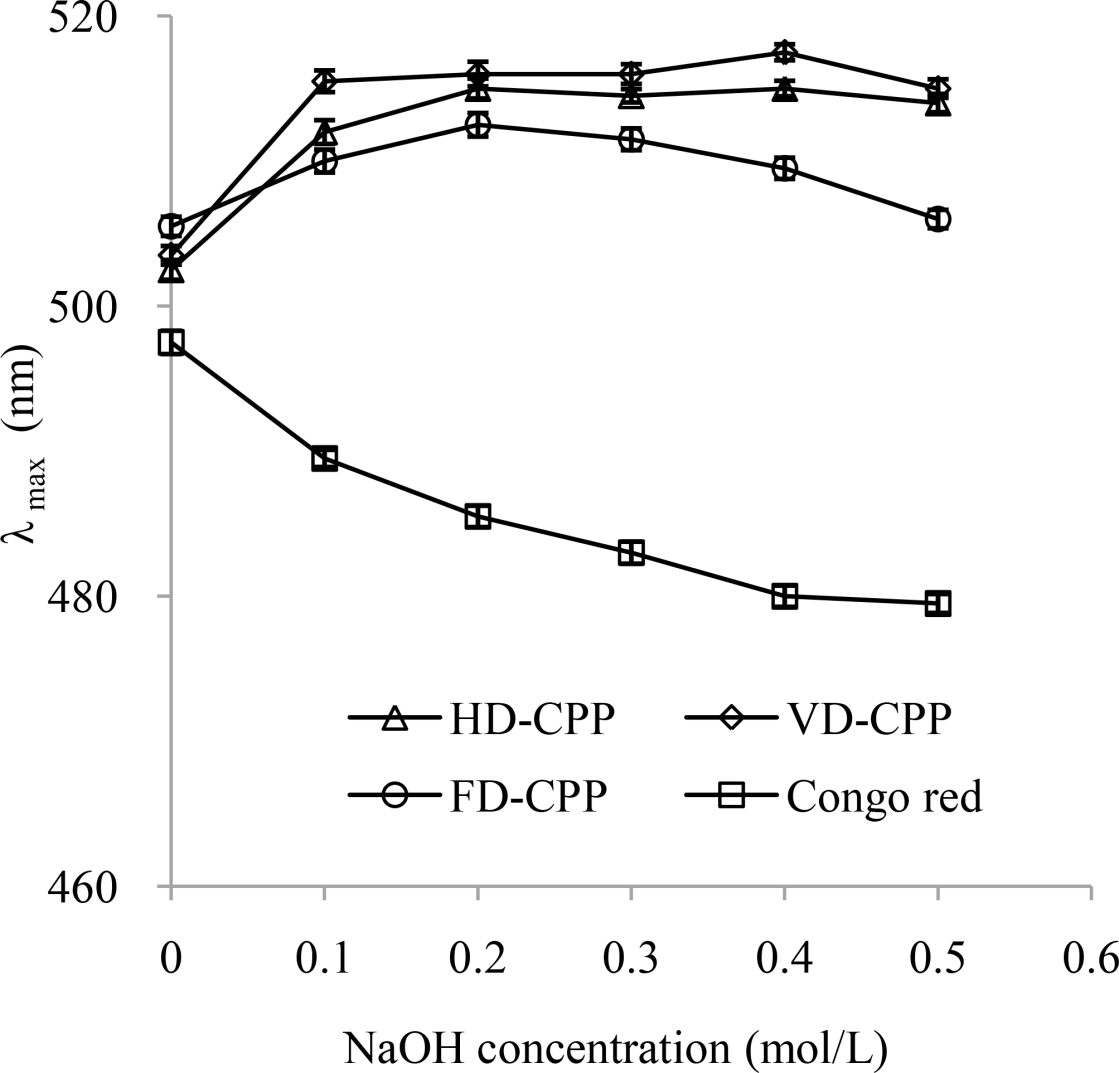
The maximum absorption of Congo red-CPP complexes at different concentrations of NaOH solution. CPP, polysaccharide from cup plant; HD-CPP, polysaccharide obtained by hot air drying; VD-CPP, polysaccharide obtained by vacuum drying; FD-CPP, polysaccharide obtained by freeze drying.

The conformation information of polysaccharide can be simply and rapidly analyzed by the Congo red assay. A biopolymer can be formed between Congo red and polysaccharide with the triple-helix conformation and exhibit a large red shift in λ_max_ compare to the Congo red solution. When the biopolymer is exposed in the alkaline condition of enough strength, the helix conformation of polysaccharide is destroyed and the λ_max_ of the biopolymer declined. The influences of NaOH concentrations on the λ _max_ of the Congo red-CPP complexes were shown in Fig 4. Comparing with the Congo red solution, the three CPP obtained by different drying methods exhibited a red shift in λ_max_, which suggested that CPP possessed the triple-helix conformation.

### Antioxidant activities of CPP obtained by different drying methods

#### DPPH radical scavenging activity

**Fig 5:**
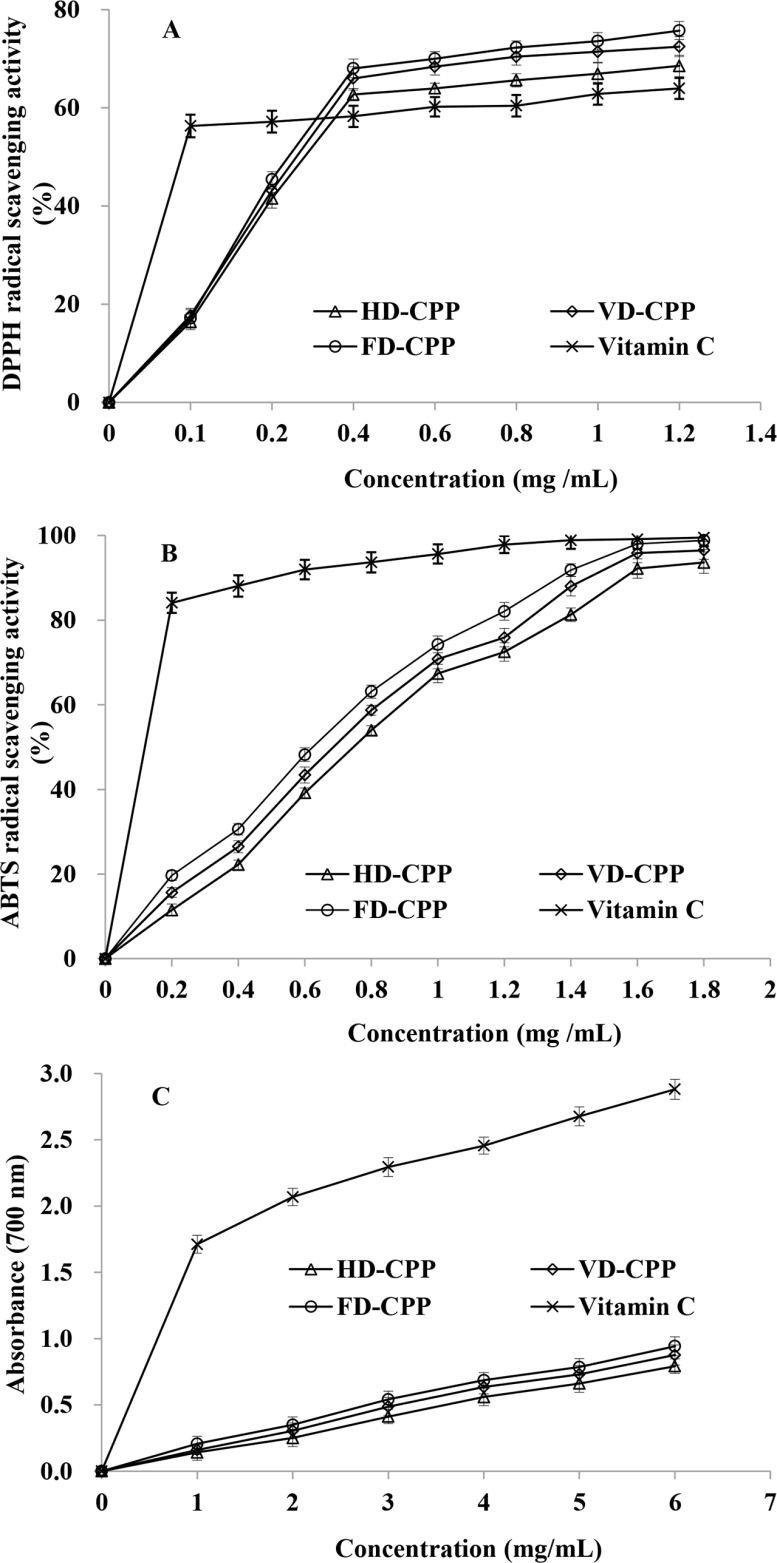
Antioxidant activities of the polysaccharide from cup plant: (A) DPPH radical scavenging activity; (B) ABTS radical scavenging activity; (C) Ferric reducing power.

The scavenging activity on DPPH radical assay has been widely used to determine the antioxidant capacity of samples due to its easy accessibility and the stability of DPPH radical [27]. The scavenging abilities of HD-CPP, VD-CPP and FD-CPP on DPPH radical were shown in Fig 5A. The results indicated the DPPH radical scavenging activities of the three CPP increased quadratically (*P*<0.05) as the concentration of polysaccharide ranged from 0.1 to1.2 mg/mL. At each concentration, FD-CPP showed higher scavenging activity against DPPH radical compared to HD-CPP and VD-CPP, and there were significant differences of DPPH radical scavenging abilities between FD-CPP and HD-CPP when the determined concentration of polysaccharide was more than 0.4 mg/mL (*P*<0.05). Taking the differences among the chemical composition (contents of total polysaccharide, protein and uronic acid) of HD-CPP, VD-CPP and FD-CPP into consideration, a conclusion can be made that different drying methods indeed affected the DPPH radical scavenging activities of polysaccharide from cup plant. The DPPH radical scavenging activities of the three CPP were higher than those of Vitamin C when the determined concentration was more than 0.4 mg/mL (*P*<0.05). For convenience of comparison, DPPH radical scavenging activity of CPP at the concentration of 1.0 mg/mL (HD-CPP 66.93%, VD-CPP 71.41% and FD-CPP 73.57%) was selected to compare with the scavenging activities of other similar botanicals or polysaccharides. At the concentration of 1.0 mg/mL, for the three drying methods including hot air drying, vacuum drying and freeze drying, the DPPH radical scavenging capacities of polysaccharides from *Inonotus obliquus* were about 40% [28]; the scavenging effects against DPPH radical of polysaccharides from *Ganoderma lucidum* were in the range of 20% to 40% [10], which indicated that CPP had strong scavenging activity against DPPH radical.

#### ABTS radical scavenging activity

The ABTS radical scavenging activity assay was shown to be simple and quick in operation, and has been extensively used to evaluate the antioxidant activity of biological samples [29]. The effects of drying methods on ABTS radical scavenging ability of CPP were shown in Fig 5B. The activities of the three CPP toward ABTS radical increased quadratically (*P*<0.05) with the CPP levels increasing. The scavenging powers of FD-CPP were significantly higher than those of HD-CPP and VD-CPP at the concentration from 0.2 to 1.8 mg/mL (*P*<0.05). The reason might be due to the higher polysaccharide yield and the higher contents of total polysaccharide, protein and uronic acid in FD-CPP. It was reported that the hydroxyl groups in polysaccharide played an important role in ABTS radical scavenging activity [10]. However, oxidation reaction of the hydroxyl groups could easily occur under the aerobic condition. The polysaccharide was dried under aerobic condition during hot air drying. Therefore, the reason that HD-CPP possessed the lowest ABTS radical scavenging activity among the three CPP might be related to the oxidation of the hydroxyl groups. There was no significant difference between ABTS radical scavenging activity of FD-CPP (98.06±2.07%) and Vitamin C (99.13±2.30%) at the concentration of 1.6 mg/mL (*P* > 0.05), as well as at the concentration of 1.8 mg/mL (*P* > 0.05). The ABTS radical scavenging activity of *Hohenbuehelia serotina* polysaccharide of freeze drying was about 30% at the concentration of 4 mg/mL [10], and the scavenging activity of polysaccharide from *Brassica rapa* L. was about 23% at the concentration of 2 mg/mL[30], which suggested that FD-CPP had significant scavenging activity on ABTS radical.

#### Ferric reducing power

Ferric reducing power could be recognized as a significant symbol to evaluate the potential antioxidant activity of natural products [31]. The ferric reducing capability of the three CPP was presented in Fig 5C. The ferric reducing powers of the three CPP showed well linear connection (*R*^2^ > 0.99, *P* < 0.05) to the test concentration. The ferric reducing power of FD-CPP was significantly higher than that of HD-CPP and VD-CPP at each concentration point (*P*<0.05). In the concentration range from 1 to 6 mg/mL, the ferric reducing power of FD-CPP increased from 0.208 to 0.943, while the reducing power of vitamin C was 1.712 at 1 mg/mL. The three CPP showed significant lower reducing power than vitamin C at the doses of all detecting concentration (*P* < 0.05). However, it was reported that the ferric reducing power of polysaccharides from garlic [32] and *Hohenbuehelia serotina* [10] were about 0.500 at the concentration of 10 mg/mL. These results indicated that FD-CPP had potential antioxidant activities of ferric reducing power.

The *in vitro* antioxidant activities assay in the present study showed that CPP had significant antioxidant activities of DPPH and ABTS radicals scavenging abilities and ferric reducing power. Actually, the antioxidant activities of polysaccharides might be based on various mechanisms. Firstly, the crude polysaccharides might contain some other antioxidants, such as peptide, flavone, pigment, polyphenol and protein, and these compounds might have contribution to the antioxidant abilities of polysaccharides [33]. Secondly, the chelating metal abilities of polysaccharides have contribution to their antioxidant abilities, and the existence of sulfate groups and uronic acid in the molecular structure of polysaccharides is essential in presenting the chelating ability of polysaccharides [34]. The protein content, uronic acid content and sulfate radical content of FD-CPP were found to be 1.14%, 3.57% and 1.81%, respectively, and these chemical compounds might have contribution to the antioxidant abilities of CPP. Thirdly, the structural features, such as molecular weight, glycosidic bond type and conformation of polysaccharides are related to their antioxidant abilities. Therefore, further research including the purification process, molecular weight, glycosidic bond type and conformation of CPP need to be studied in order to clarify the mechanism of antioxidant activities of CPP.

## Conclusions

The extractive condition of the polysaccharide from cup plant was optimized by performing RSM. The results indicated that the test variables of water to raw material ratio, extraction time and extraction temperature had significant effects on the extraction yield of CPP (*P* < 0.05). Quadratic regression model was used to predict responses and it had satisfactory fit to the actual values. A maximum yield of CPP of 6.49% was obtained under liquid–solid ratio 15 mL/g, extraction time 61 min, and extraction temperature 97°C. In addition, different drying methods had significant effects on the antioxidant activities and physicochemical properties of CPP such as the chemical composition (contents of total polysaccharide, protein and uronic acid), relative viscosity and solubility. Compared with the CPP obtained by hot air-drying and vacuum drying, CPP obtained by freeze drying presented significant higher ferric reducing power and scavenging activities against DPPH and ABTS radicals (*P* < 0.05). The results suggested that freeze drying method served as a good method for the preparation of polysaccharide from cup plant. Further works on the antioxidant activity mechanism of CPP are in progress.

## Supporting information

S1 Dataset Single-factor experiments for CPP extraction. (XLS)

S2 Dataset Solubility of CPP. (XLS)
